# Contrasting Effects of Climate Change on Rabbit Populations through Reproduction

**DOI:** 10.1371/journal.pone.0048988

**Published:** 2012-11-13

**Authors:** Zulima Tablado, Eloy Revilla

**Affiliations:** 1 Estación Biológica de Doñana, Consejo Superior de Investigaciones Científicas, Sevilla, Spain; 2 Laboratoire d’Ecologie Alpine, Centre National de la Recherche Scientifique, Bourget du Lac, France; 3 Northern Institute for Nature Research, Tromsø, Norway; University of Hong Kong, Hong Kong

## Abstract

**Background:**

Climate change is affecting many physical and biological processes worldwide. Anticipating its effects at the level of populations and species is imperative, especially for organisms of conservation or management concern. Previous studies have focused on estimating future species distributions and extinction probabilities directly from current climatic conditions within their geographical ranges. However, relationships between climate and population parameters may be so complex that to make these high-level predictions we need first to understand the underlying biological processes driving population size, as well as their individual response to climatic alterations. Therefore, the objective of this study is to investigate the influence that climate change may have on species population dynamics through altering breeding season.

**Methodology/Principal Findings:**

We used a mechanistic model based on drivers of rabbit reproductive physiology together with demographic simulations to show how future climate-driven changes in breeding season result in contrasting rabbit population trends across Europe. In the Iberian Peninsula, where rabbits are a native species of high ecological and economic value, breeding seasons will shorten and become more variable leading to population declines, higher extinction risk, and lower resilience to perturbations. Whereas towards north-eastern countries, rabbit numbers are expected to increase through longer and more stable reproductive periods, which augment the probability of new rabbit invasions in those areas.

**Conclusions/Significance:**

Our study reveals the type of mechanisms through which climate will cause alterations at the species level and emphasizes the need to focus on them in order to better foresee large-scale complex population trends. This is especially important in species like the European rabbit whose future responses may aggravate even further its dual keystone/pest problematic. Moreover, this approach allows us to predict not only distribution shifts but also future population status and growth, and to identify the demographic parameters on which to focus to mitigate global change effects.

## Introduction

Anthropogenic climate change is affecting many physical and biological processes worldwide [1], and investigating its impact on populations and distributions of vulnerable or invasive species is one of the main research priorities [2], [3]. Previous research has mostly used climate conditions within present geographical ranges to predict future species distributions and population responses without considering the underlying mechanisms [4], [5]. However, given the potential complexity of interactions between climatic variables and population parameters, in order to make more reliable large-scale predictions we should first understand how climate change will alter the individual processes driving population dynamics and distribution [6], [7]. Here, we focused on the duration of the reproductive period as model of demographic trait to investigate the mechanisms through which climate change may alter large-scale patterns of population dynamics of a species such as the European rabbit (*Oryctolagus cuniculus*). The relevance of this species resides in that is both a keystone species in its native range (SW Europe), where it plays a crucial role as ecosystem engineer, prey for predators and game species [8], [9], [10], and an invasive pest in other areas.

Since rabbits are “fast” mammals [11], their conservation status and success will substantially depend on its high breeding potential. This fact together with the well-known direct link between rabbit reproduction and environmental factors such as climate, photoperiod, and food availability [12], [13], [14], provide us with an ideal study system to address our objective. We combined an existing mechanistic model, which efficiently predicts breeding season months based only on temperature, precipitation and photoperiod [14], and a rabbit demographic model we developed in order to explore the impact of climate change on future breeding seasons and population trends across large scales. Studies like this focusing on the underlying mechanisms to explain and predict population responses will be essential to better understand future consequences of the undergoing global change and to help develop adequate mitigation strategies.

## Materials and Methods

We followed a two-step approach to understand the effect of climate change on rabbit populations through the modification of their breeding season. First, we used a mechanistic model which classifies correctly over 83% of breeding season months anywhere within rabbit’s current distribution around the world based on average day length, photoperiod change, a simple and quadratic effect of monthly temperature, and an index of green pastures based on the evapotranspiration of previous months [14]. Secondly, to determine the consequences at the population level of these changes in breeding season, we built and parameterized a population dynamics model according to the worldwide information about rabbit reproduction and mortality gathered in ref. [14] and ref. [15], and used it to simulate how rabbit populations responded under different breeding season scenarios.

### Predicting Climate Driven Changes in Breeding Season

Applying the mechanistic model in ref. [14] to the monthly climate projections across Europe (at 50 km resolution) for a control (1961–1990) and future (2071–2100) period, which are available from the Rossby Centre Regional Atmosphere-Ocean model (RCAO) [16], we were able to examine large scale effects of climate change on length and interannual variability (CV) of rabbit breeding season. The RCAO regional model was developed within the PRUDENCE project ([17]; http://prudence.dmi.dk/). It was driven by boundary conditions taken from two different general circulation models (GCM) [17], [18]: HadAM3H (Hadley Centre Atmospheric Model version 3H) [19], and ECHAM4/OPYC (European Centre Hamburg model 4) [20]. Within each GCM we also considered two different scenarios of future greenhouse gas emissions [21]: A2 (high) and B2 (moderate). Since rabbits do not breed below 0°C, reproduction was directly assumed to be absent from months with those temperatures. Finally, complementary photoperiod data necessary to predict breeding season according to ref. [14] were obtained from the US Naval Observatory (http://www.usno.navy.mil/USNO/astronomical-applications/data-services/rs-one-day-world).

### Modelling Rabbit Population Dynamics

#### Demographic model description

**Figure 1:**
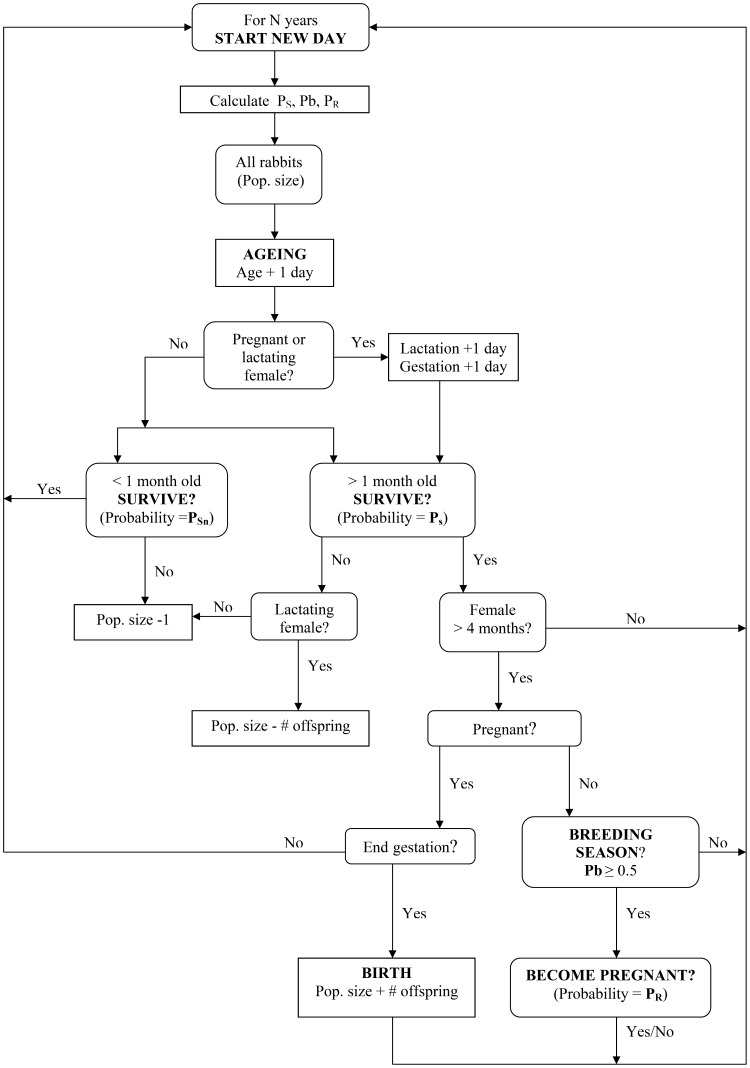
Diagram showing the structure of the population dynamics model for European wild rabbits.

We developed a non-spatial stochastic individual-based model representing a wild rabbit population in an area of 5 hectares. It includes the basic life cycle in which the gestation lasts for 30 days, newborn rabbits stay in their warrens and depend on their mother up to day 30, after which they are weaned, juvenile rabbits reach sexual maturity at 4 months old and rabbit longevity is 6.8 years [14], [15], [22]. Model runs at daily steps updating individual rabbit states as necessary according to the population diagram (Fig. 1). Populations are started with N rabbits of: 1) random gender, 2) age randomly selected from a Gaussian distribution with mean = 515 days and sd = 162 and truncated at three standard deviations, which restricts ages to the range between 30 and 1000 days approximately, which is a reasonable life expectancy in wild rabbit populations [22], [23] and 3) if female, absence of pregnancy, lactation or offspring.

#### i. Reproduction (Table 1; Fig. 1)

Rabbits are only allowed to breed in months within the reproductive period which is delimited by climate, food availability (i.e. index obtained from temperature and precipitation in previous months) and photoperiod according to the following equation from ref. [14]:
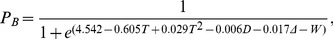
(1)


**Table 1:** Parameters of the rabbit population model.

PARAMETER DESCRIPTION	SYMBOL	VALUES
Population density	*η*	
Carrying capacity	*K*	
Rabbit age (in days)	*A*	
Reproduction		
Probability of being within the a breeding season	*P_B_*	(Eq. 1)
Mean monthly temperature (°C)	*T*	Meteorological station data
Average daylength in a month (light minutes/day)	*D*	Meteorological station data
Photoperiod change between 2 consecutive months	*Δ*	Meteorological station data
Availability of green pastures in a given month	*W*	= 0 if in both previous months, precipitation <2*Temperature
		= −1.592 otherwise (Ref. [14])
Breeding probability of mature females	*P_R_*	(Eq. 2)
Age effect on female fecundity	*r_A_*	*r4* if female age = 4–6 months
		*r4+ r6* if female age = 6–9 months
		*r4+ r9* if female age >9 months
Factor of density-dependence in reproduction	*dr*	
Average litter size	*L*	3.2–7.3 rabbits/litter (Ref. [24], [25])
Monthly proportion of pregnant females	*B_(A)_*	(Eq. 3)
Survival		
Daily survival probability of newborns (up to 30 days)	*P_SN_*	(Eq. 4)
Monthly newborn mortality	*M_N_*	0.4–0.9 (Ref. [26], [27], [28])
Daily survival probability of rabbits older than 30 days	*P_S_*	(Eq. 5)
Age effect on survival	*δ_A_*	*δ4 - δ1* if rabbit age = 1–4 months (juveniles)
		*δ4* if rabbit age >4 months (adults)
Factor of density-dependence in survival	*ds*	
Effect of food availability on survival	*ν*	
Number of consecutive dry months	*F_m_*	
Monthly output survival rate	*S_(A)_*	(Eq. 6)

where *P_B_* is the probability of being within the breeding season (i.e. reproduction will occur in months in which *P_B_* is ≥0.5), *T* is the mean monthly temperature, *D* is the average day length in each month, Δ is the difference between the *D* of a month and the *D* of the previous month, and *W* represents food availability (*W = *0 if previous two months were dry, that is, precipitation <2*temperature, and *W = *−1.592 if precipitation was higher than twice the temperature in at least one of the previous two months). More details about the source of the numerical coefficients can be found in reference [14].

Within the breeding season, sexually mature female rabbits (i.e. over 122 days), which are not already gestating, will have a probability of becoming pregnant (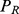) that will depend on their age and population density as shown by Equation 2:
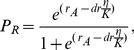
(2)where *r*_A_ is a parameter whose value increases with age class (4–6 months, 6–9 months and over 9 months) and plays the role of the intercept term, and 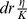 is the factor representing density dependence in reproduction (with *d_r_* being a constant, *η* the population density and *K* the carrying capacity). If pregnancy occurs, gestation lasts 30 days. Resultant monthly proportions of pregnant females 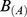 of each mature age class (*A*; 4–6 months, 6–9 months and over 9 months) are then calculated as
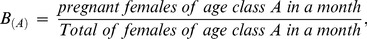
(3)When the gestation period is over, females give birth to a number of kittens chosen randomly from Gaussian distribution with mean equal to the average population litter size (L) and the extremes truncated at two standard deviations. This standard deviation is set to one according to similar literature values. Then, females enter the lactation period (30 days) in which newborns depend on them. Due to post-partum oestrus females might become pregnant the day after giving birth with a probability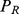. Newborn will be recruited into the population with an age of 0 days and random gender. If they turn to be females they will be neither pregnant nor lactating.

#### ii. Mortality (Table 1; Fig. 1)

European wild rabbits have high mortality rates which decrease with age. Main causes of death are predation and endemic diseases, that is, myxomatosis and viral haemorrhagic disease. Burrow flooding and collapse, road kills, hunting and other diseases (e.g. coccidiosis) might also contribute to population regulation.

Newborn rabbits (less than one month old) which are highly associated to breeding stops and warrens present the higher mortality rates. The daily probability of survival of young rabbits less than one month of age is given by:
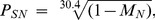
(4)where 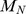 is the monthly mortality rates for newborn in wild rabbit populations and 30.4 is the average month length in days. Death of suckling females will imply also the loss of their offspring contributing to reach the mortality figures (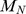) reported by the authors.

Since juvenile and adult compete for food resources, refuge and warrens, the daily probability of survival (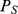) will decrease as population density increases, and as the number of months with reduced food resources augments based on the equation.
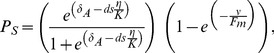
(5)where 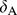
is a parameter acting as the intercept of the function and which varies according to age class (juveniles: from 1–4 months and adults: over 4 months), 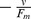 is a term representing food scarcity (with 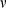 being a constant and 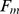 the number of consecutive dry months (precipitation <2*temperature) until month *m,* and 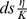 accounts for the density-dependence in survival (where 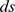 is a constant, η is the population density and K the carrying capacity). The monthly output survival rates for the simulated population are then given by
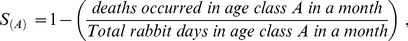
(6)where age A is classified as newborn, juveniles and adults.

#### Sensitivity analyses of the model

We performed sensitivity analyses to investigate the response of the population dynamics model to variations in the different input parameters. Values and ranges for the parameters of the model (Table 2) were obtained, when possible, from the information reviewed in ref. [14] and [15] (e.g. litter sizes or breeding season equation terms). Breeding season months were established according to conditions in Mediterranean ecosystems (e.g. Southern Spain), from where the species is native. Note that parameters of the function predicting breeding season (Eq. 1) were already estimated and validated [14], and thus, are not subject to variation in our model.

**Table 2:** Input parameter ranges used in the sensitivity analyses (Latin Hypercube sampling).

Parameters	Description	Range	Source
Reproduction			
*P_R_* (Eq. 2)*	Daily probability of fecundation		
*r_A_*	Age effect on female fecundity		
	r4 (for rabbits from 4 to 6 months)	[−3, 2]	
	r6 (for rabbits from 6 to 9 months)	[2], [4]	
	r9 (for rabbits over 9 months)	[4.5, 7]	
*Dr*	Factor of density-dependence in reproduction	[0.2, 10]	
*L*	Mean litter size	[3.0, 7.4]	Ref. [24], [25]
**Survival**			
*M_N_*	Monthly newborn mortality (up to 30 days old)	0.4–0.9	Ref. [26], [27]
*P_S_* (Eq. 5)*	Daily probability of survival for rabbits over 30 days		
*δ_A_*	Age effect on survival		
	δ_1_ (for juvenile rabbits; from 1–4 months)	[2], [4]	
	δ_4_ (for adult rabbits; over 4 months)	[5], [10]	
*Ν*	Effect of food availability on survival	[10, 50]	
*Ds*	Factor of density-dependence in survival	[0.2,10]	

* *Range of parameter values are adjusted according to real data of populations all over the world (Ref.* [*[14]* *,* *[15]* *,* *[29]* *).*

For the rest of the model, such as our own defined fecundity and survival functions, the parameter space was constrained by imposing that patterns emerged from the simulated data agreed with patterns observed in wild populations. That is, we only considered parameter values that led to seasonal fluctuations, pregnancy and survival increasing with age, and reproductive and survival rates falling within the ranges reported by ref. [14] and [15] for real wild populations worldwide.

Latin hypercube sampling (LHS) [30], [31] was applied in order to sample the ranges of the different parameters in the model and, thus, select the combinations of input values for the sensitivity analyses. In LHS the range of each parameter is divided into *n* non-overlapping equiprobable intervals. A random number is used to select (without replacement) one of the intervals at a time and the central value of that interval is then taken following the lattice sampling technique [31], [32], which is a special case of LHS. The advantage of using LHS in sensitivity analyses is that, since samples are more evenly distributed throughout the parameter ranges, we are able to cover more efficiently all regions of the parameter space with fewer trials [33], [34], [35].

With this technique we end up with *n*-sized samples for each parameter which are then randomly arranged to generate a set of *n* parameter combinations (in this study *n* = 5). We repeated this process a total of 1000 times. We then ran the simulation model for each of these parameter combinations (n*1000) to produce a set of output observations. Each simulation was also replicated 50 times and the results of the replicates were averaged before further analyses. All simulations ran for 10 years and initial population density was set at a level that was moderately high (i.e. 14 rabbits/ha) to minimize initial stochastic population collapses, but that, at the same time, was equal to the carrying capacity to favour early population stability.

We then applied generalized linear models (GLMs) to the simulation results to examine the impact of varying input parameters on final simulated population sizes. We used probability of extinction and mean population size after ten simulated years as the dependent variables with beta error distribution (link function = logit) and negative binomial distribution (link function = log) respectively. In the case of extinction probabilities, values had to be slightly transformed by adding a small constant (i.e. 0.0001) to eliminate zero values and allow the fitting of a beta distribution. The standardized coefficients estimated in this way were then used to determine relative importance of each model parameter on the simulated population dynamics.

The results of these GLMs indicated that the probability of extinction seems to be sensitive mostly to changes in survival parameters, especially to the adult age parameter (i.e. survival specific of adults) and food availability factor (Table 3). Final population size was also mainly sensitive to survival parameters and to a lesser extent to reproductive parameters (Table 3), with the highest standardized coefficients being shown by the density-dependence factor and adult survival parameter, followed by density dependence in the fecundity function and the food factor.

**Table 3:** Results of the sensitivity analysis.

Effect	Probabilityof extinction	Meanpopulation size
i. Model input parameters	Std. Coef a	Std. Coef b
Daily probability of fecundation (Eq. 2)		
Age parameter (from 4 to 6 months old)	(−0.89) ^ns^	4.64
Age parameter (from 6 to 9 months old)	(0.54) ^ns^	(−0.45)^ ns^
Age parameter (over 9 months old)	(0.54) ^ns^	1.96
Density-dependence in fecundity	(1.02) ^ns^	−20.18
Mean litter size	(−0.96) ^ns^	4.28
Newborn probability of mortality	4.23	−9.34
Daily survival probability over 30 days old (Eq. 5)		
Age parameter (juveniles; from 1–4 months)	5.41	−9.88
Age parameter (adults; over 4 months old)	−15.91	36.28
Food availability factor	−22.86	17.86
Density-dependence in survival	14.23	−55.14
ii. Rabbit life history traits	Std. Coef c	Std. Coef d
Mean litter size	−16.85	42.00
% of pregnant females (4–6 months)	(0.06) ^ns^	(−1.07)^ ns^
% of pregnant females (6–9 months)	(−0.45) ^ns^	−18.95
% of pregnant females (over 9 months)	−41.19	113.78
Newborn survival rates	−25.04	62.93
Juvenile survival rates	−9.09	53.71
Adult survival rates	−61.12	104.00

Generalized linear models for the effect of (i) model parameters and (ii) rabbit life history traits on mean population size (error distribution = negative binomial, link = log) and extinction probability (error distribution = beta, link = logit) after 10 years. The magnitude of the standardized coefficients (Std. Coef) represents the relative importance of each explanatory variable. Population dynamics will be more sensitive to parameters showing larger absolute values.

() *ns = non-significant.*

a *Goodness of fit (GoF = Pearson Chi-Square/DF) = 0.7;*

b *GoF = 1.4;*

c *GoF = 0.8;*

d *GoF = 3.3.*

We performed additional analyses to investigate the sensitivity of the model to life history traits usually described for wild populations in the literature (e. g. pregnancy rates and survival). Thus, we repeated the previous GLMs, but by using as explanatory variables the realized survival and pregnancy rates for the different age classes obtained from the simulated populations, together with the effect of input litter sizes (Table 2). These analyses greatly confirmed the outcome of the previous ones (Table 3) and showed that extinction risk appeared to be mostly sensitive to variations in adult and newborn survival rates, and in the percentage of pregnant females over 9 months old. Pregnancy rates of older females also proved to be important for the mean number of rabbits since they caused the highest population size sensitivities, followed by survival rates. Rabbit population dynamics were least sensitive to variation in pregnancy percentages of the younger age classes (Table 3).

Sensitivity GLMs and further statistical analyses of this study were all performed using the program SAS (SAS Institute Inc., Cary, NC, USA) and in every analysis we used the error distribution that best fitted to the data. Their goodness of fit (GoF) was assessed from the ratio of the obtained generalized chi-square to the degrees of freedom. The closer the ratio is to one the better the model described the variability of the data [36].

#### Rabbit population dynamics under different breeding season scenarios

The individual-based model was later used to perform simulations of rabbit populations under different 30-year scenarios of breeding season length and CV (Table 4). Among the thousands of local breeding seasons predicted for all Europe (i.e. resolution of 50 km) we chose 12 cases which were representative of all the extent of possible reproductive periods. The benefit of running the simulations on a subsample of possible breeding seasons rather than on all locations across Europe is that we are still able to identify general large scale trends while considerably reducing the time of execution (over 100 times faster). Within each scenario we also varied the parameters identified as most important by the sensitivity analyses (Table 3 and 4). The other model parameters (e.g. litter size and fecundity function terms) were assigned an average value (Table 4). Note that given the covariation among climate, breeding season and index of green pastures, the latter had to be varied according to the scenario and climatic conditions tested in each case. As in the case of sensitivity analyses, initial density was set at carrying capacity to increase population stability and Latin Hypercube sampling with Lattice sampling technique [30], [31] was applied in order to select the 5*1000 combinations of parameters for the simulations, which were also replicated 50 times and had their results averaged before further analyses.

**Table 4:** Parameter values and breeding season scenarios used to model rabbit population dynamics.

i. Model input parameters	Population dynamics		Response time	
Daily probability of fecundation (Eq. 2)				
Age parameter (from 4 to 6 months old)	0		0	
Age parameter (from 6 to 9 months old)	3		3	
Age parameter (over 9 months old)	5.5		5.5	
Density-dependence in fecundity	1.5, 5		2	
Mean litter size	5		5	
Newborn probability of mortality	0.4–0.8		0.4–0.8	
Daily survival probability over 30 days of age (Eq. 5)				
Age parameter (juveniles; from 1–4 months)	2, 4		2, 4	
Age parameter (adults; over 4 months old)	5–10		5–10	
Food availability factor	15, 40		15, 40	
Density-dependence in survival	1.5, 5		2	
Carrying capacity	14		14, 28	
Initial population density	14*		1	
**ii. Breeding season scenarios**	**Population dynamics**		**Response time**	
	**Length(months)**	**CV(%)**	**Length(months)**	**CV(%)**
Scenario 1:	5.45	16	5.45	16
Scenario 2:	3.55	24.5	3.55	24.5
Scenario 3:	9.72	14.5	9.72	14.5
Scenario 4:	8.17	8.7	8.17	8.7
Scenario 5:	6.90	14.7	6.90	14.7
Scenario 6:	8.38	8.1	8.38	8.1
Scenario 7:	4.14	8.5	4.14	8.5
Scenario 8:	5.48	13.5	5.48	13.5
Scenario 9:	8.31	11.4		
Scenario 10:	6.90	12.8		
Scenario 11:	8.38	7.9		
Scenario 12:	9.55	8.5		

Values under the *Population dynamics* heading were used for testing the effect of length and variability of breeding season on population size and extinction probability, while the *Response time* ones refer to analyses examining the potential rate of increase of rabbit populations after perturbations causing collapses. Breeding season scenarios consist of a mean (in months) and inter-annual variability (Coefficient of Variation in %) over a 30-year period of the duration of breeding season. These scenarios were aimed to be representative of the whole range of possibilities found in real populations worldwide.

* *Initial density was set at carrying capacity to increase population stability.*

The output of these simulated populations were statistically analysed using GLMs. We tested for the impact of the two breeding season properties (i.e. length and CV of scenarios) on rabbit population dynamics and their relative importance with respect to the other demographic parameters. As dependent variables we used both final population size (with error distribution = negative binomial and link function = log) and extinction probability (with error distribution = binomial and link function = logit). The outcome of these GLMs were subsequently applied to the previously predicted reproductive periods across Europe to obtain a spatially explicit representation of the large-scale effects of climate change on rabbit population dynamics through reproduction. The effects of the other demographic terms of the GLMs (Table 5) were averaged so as to clearly show the consequences of breeding season changes.

**Table 5:** Impact of breeding season on population dynamics.

Effect	Probability of extinction^a^	Mean population size^b^
	Odds ratio(±SE)* Rank	Estimate(±SE)* Rank
Reproductive period		
Mean duration (in months)	0.44±0.018 2	1.23±0.004 4
Coefficient of Variation (in %)	1.73±0.017 1	0.90±0.001 3
Density-dependence in fecundity and survival	1.39±0.028 5	0.72±0.001 1
Newborn probability of mortality	8.25±2.449 7	−0.57±0.03 6
Daily survival probability over 30 days of age		
Age parameter (from 1–4 months old)	1.75±0.071 6	0.79±0.004 5
Age parameter (over 4 months old)	0.63±0.013 4	1.35±0.003 2
Food availability factor	0.91±0.003 3	1.01±0.0003 7

Effects of breeding season length and variability, and other model parameters on extinction probability (error distribution = binomial, link = logit) and mean size (error distribution = negative binomial, link = log) of rabbit populations at the end of a 30-year period. The relative importance of each parameter is given by the numerical order in the rank column. For further interpretation of odds ratios and coefficients (i.e. mean effect estimates) refer to parameter magnitudes found Table 4.

* *All coefficients are significant at p<0.0001.*

a *Goodness of fit (GoF = Pearson Chi-Square/DF) = 0.8;*

b *GoF = 0.8.*

Finally, since it could be argued that, despite the continental trends observed, at local scale the effect of the breeding season is neglectable with respect to survival variability resultant from epidemics [37] or extreme weather events [38], [39], we performed further simulations to prove the importance of reproductive period length and variability in population resilience. We investigated the role of breeding season in determining population recovery after a collapse (i.e. time to reach carrying capacity) by running the stochastic individual-based population model again for a range of breeding seasons (Table 4) but under reduced initial density, moderately low density-dependence, and two different levels of carrying capacity (Table 4). Thus, we simulated the response of populations after perturbations leading to important population reductions. Once more, the results of these simulations were replicated, averaged and later analysed through GLMs with response time as dependent variable (negative binomial distribution, link function = log) and carrying capacity as random factor.

## Results

### Climate Driven Changes in Rabbit Breeding Season

**Figure 2:**
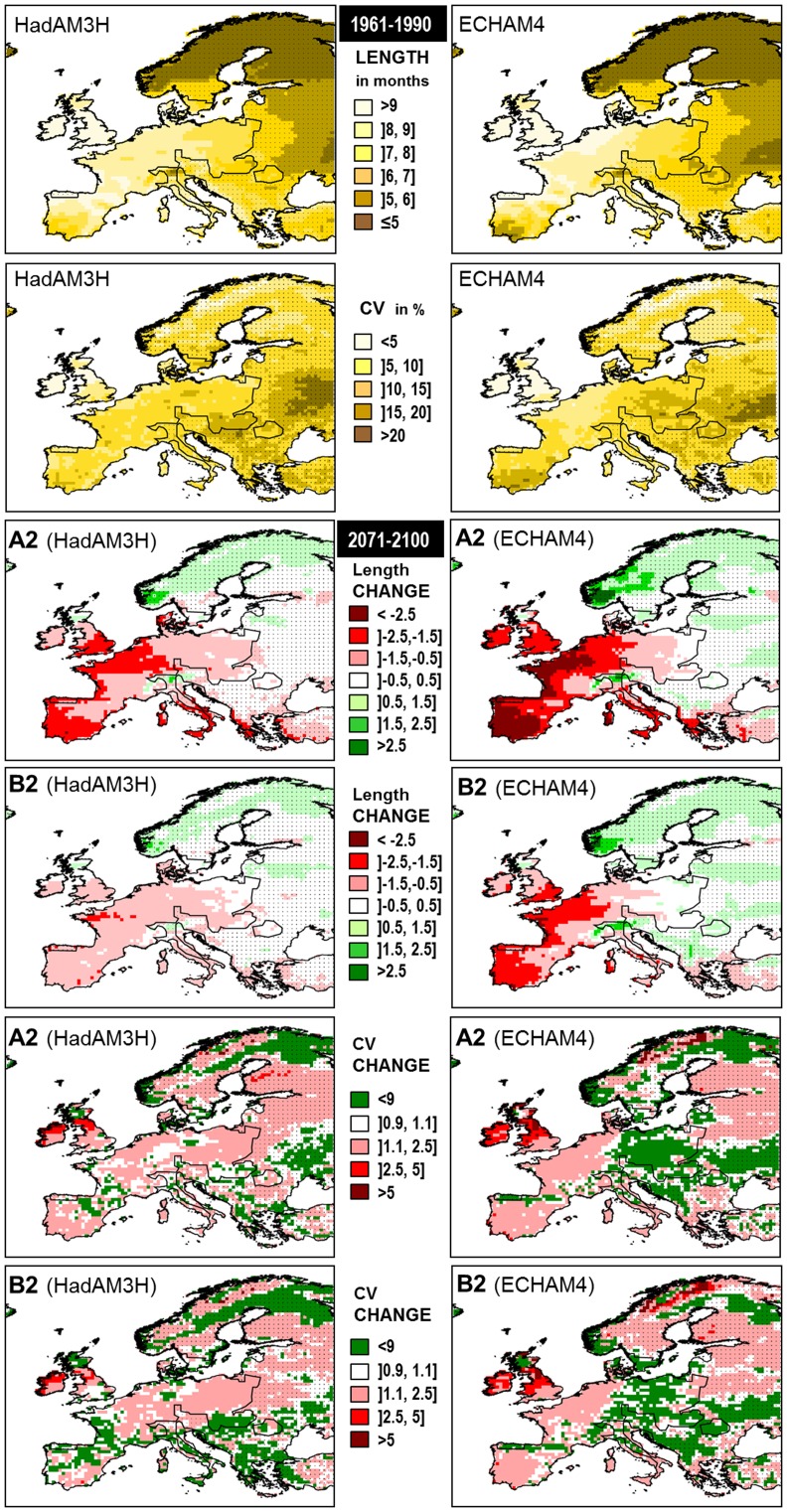
Predicted breeding season patterns across Europe based on climatic projections from the RCAO regional model. The top four Figures show the mean length (months) and coefficient of variation (CV, in %) for the control period (1961–1990). Lower Figures represent difference (in months: <1 (decrease), = 1 (unchanged) and >1(increase)) and changes in inter-annual variability (CV_future_/CV_control_: <1 (decrease), = 1 (unchanged) and >1(increase)) from control to future climate according to two different GCM (HadAM3H and ECHAM4/OPYC) and gas emission scenarios (A2 = High and B2 = Moderate). Areas outside current rabbit distribution are marked with dots.

Although there are some variations depending on the boundary conditions set by different general circulation models (GCM) or on the gas emission scenarios applied, the overall predicted regional trends are consistent. For 1961–1990 we observed a non-trivial pattern in which breeding seasons are shortest in SW Europe (i.e. Southern Iberian Peninsula) and increase in the north and east directions. Towards the most eastern longitudes of rabbit range, as well as at high alpine altitudes, colder climates reduce again the duration of the reproductive period (Fig. 2 and see figure S1). We found that inter-annual variability (CV) in the breeding season length also differs greatly from higher values in Mediterranean and continental regions, such as Southern Iberian Peninsula and Eastern Europe, to less variable seasons in the Great Britain, Ireland and mainland areas under a strong Atlantic influence (Fig. 2 and see figure S1). When comparing the control period to the projections for 2071–2100, we observed that climate change will decrease the duration and increase the variability of reproductive periods over most of the current range of rabbits in Europe, especially in the Iberian Peninsula (Fig. 2 and see figure S1). Contrarily, towards higher latitudes (i.e. Scandinavian Peninsula and Northern UK), higher altitudes (e.g. the Alps) and Eastern limits of rabbit distribution, breeding seasons will increase and become more stable (Fig. 2 and see figure S1).

### Population Responses to Breeding Season Variation

**Figure 3:**
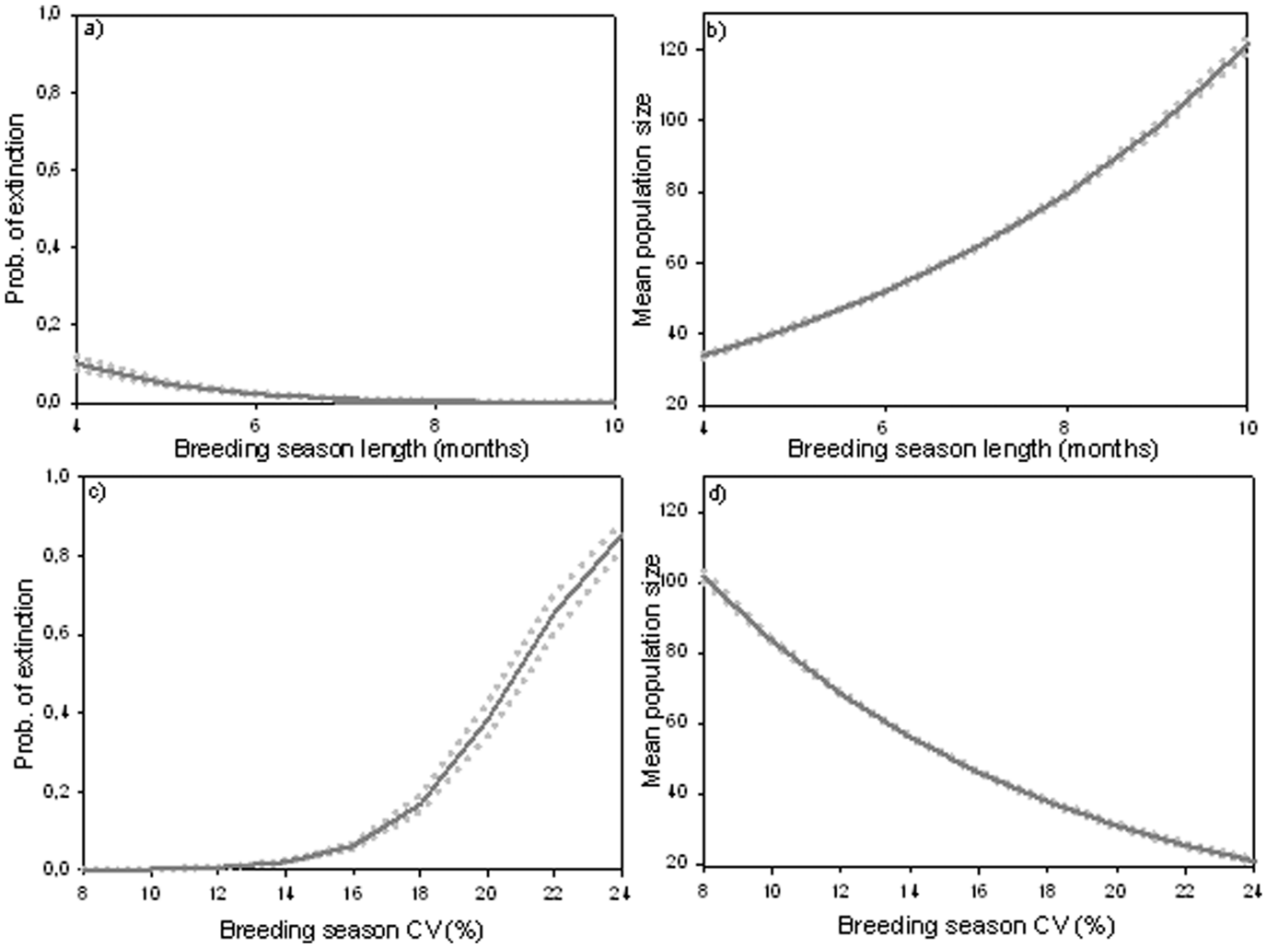
Effect of breeding season on population dynamics. a) and c) show the relationship between population extinction risk and breeding season length and coefficient of variation respectively for an averaged effect of the other variables of the GLM. In the same way b) and d) describes how mean size of rabbit populations change as we vary the duration of the reproductive period and its interannual variability respectively. Solid lines represent the average effect while dotted lines delimit the 95% confidence interval.

The sensitivity analyses of the results of simulating rabbit population dynamics under different breeding scenarios showed that large-scale variability in breeding season was the main factor driving extinction probabilities, followed by the mean duration of the reproductive period and the food availability factor (Table 5). To a lesser extent the characteristics of the breeding season were also important in determining mean population size, although in this case the density-dependence parameter and the adulthood parameter of the survival function showed the highest higher influences (Table 5; Fig. 3). Note that overall population size may change dramatically without modifying carrying capacity due to the overshooting produced by the strong seasonality of rabbit demography. As breeding season becomes shorter and more variable rabbit populations get smaller and more prone to extinction (Fig. 3). Lower values of the food availability parameter (i.e. greater negative impact of food scarcity on survival) also lead to higher extinction probabilities whereas population sizes also decrease with smaller values of the adult survival parameter and with higher density-dependence.

**Figure 4:**
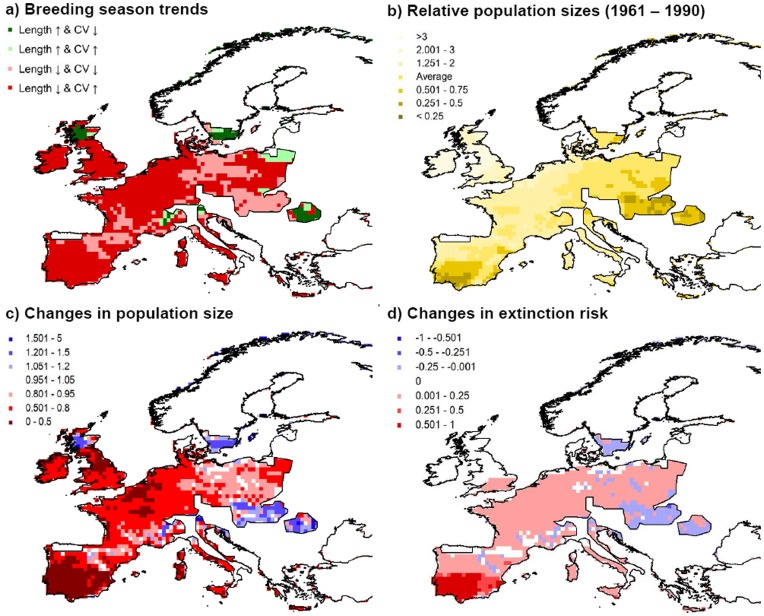
Climate change influence on population dynamics across current rabbit distribution. a) Future trends in breeding seasons calculated using ensembles of both GCM and gas emission scenarios. b) Pattern of relative population sizes (population size (N) in each cell/overall mean population size) for the control period. c) Variations in population numbers with climate change (N_future_/N_control_). d) Future changes in extinction probabilities of populations (Prob_future_-Prob_control_).

By applying the outcome of these GLMs to previous spatially-explicit predictions of breeding season change across rabbit distribution in Europe, we were able to visually represent the effects of climate change on rabbit population dynamics at large scales (Fig. 4). We observed that, mirroring alterations in reproductive periods (Fig. 2), rabbit numbers will decrease in most of the rabbit range especially in areas where not only the length of the reproductive period will decrease but also become more variable. The strongest declines will occur in the South of the Iberian Peninsula where, in addition, predicted population sizes are already low in the control period (Fig. 4). On the other hand, towards the eastern and northern borders of the species current distribution, mean population numbers are expected to increase and extinction risks to decrease, principally in regions where breeding season will both increase and stabilize. We would like to emphasize that although we used the GLM coefficients, which summarize the simulation results, as a simplification allowing us to spatially represent the trends, the interpretation of the results does not only come from the graphic representation but are mostly based on the results of simulating a range of different climate scenarios.

**Figure 5:**
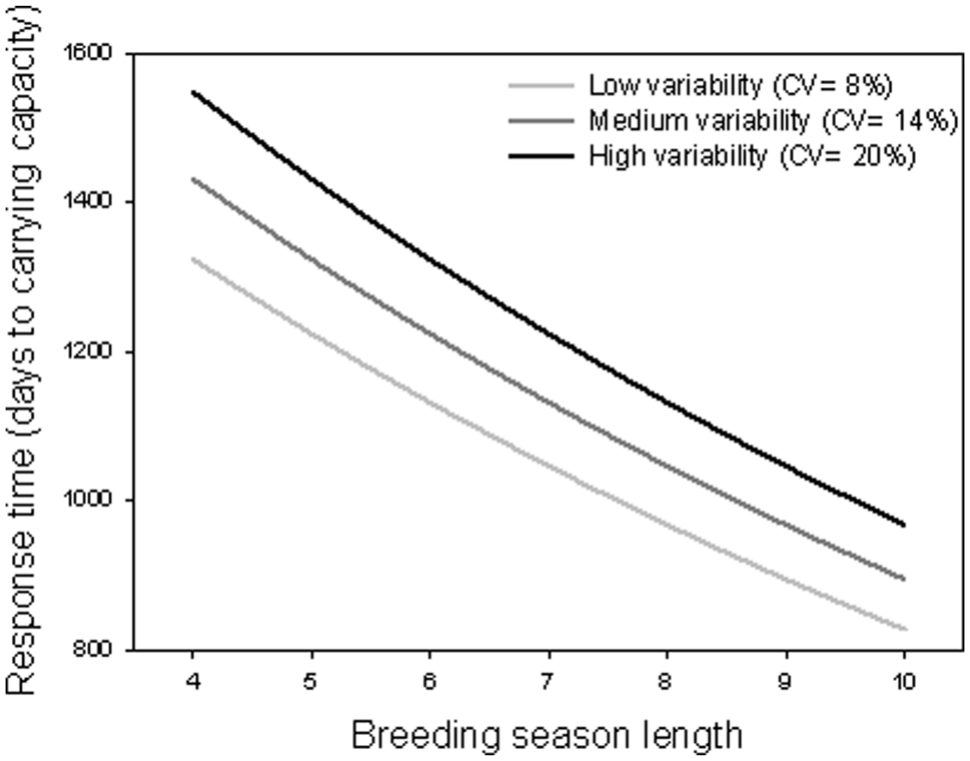
Association between the capacity of populations to recover after collapses and the length and CV of their breeding season. Reduction in the population response time (days necessary to reach carrying capacity again) with increasing duration and decreasing interannual variability of the breeding season.

As for the capacity of local populations to recover after a collapse, the statistical model (GLM; Goodness of fit = 1.0) showed that in situations such as this, where carrying capacity is not limiting, populations increase significantly faster if they have longer reproductive periods regardless of the variation in the other population parameters (F = 165.52, p<0.0001; Fig. 5). Inter-annual variability also correlates importantly with the response rate; however, its effect is negative and less strong than in the case of season length (F = 30.91, p = 0.0001; Fig. 5).

## Discussion

Our findings show the diversity of effects that climate change may have on a same species through the modification of only one of its main demographic parameters. We found that despite the slight differences among the general circulation models (GCM) or the gas emission scenarios used [40], [41], there exist strong patterns revealing the high impact of climate change on rabbit breeding season. Future reproductive periods will tend to decrease in magnitude and increase in variability in most of the current European distribution of this species. This negative effect will be especially worrying towards the SW of Europe, where seasons were already moderately short in the control period as shown by our predictions for 1961–1990, which at the same time agree to a large extent with published data from real populations (see Table S1 and Text S2). In fact, predictions using climate data of Lisbon (West of the Iberian Peninsula) for the last century show that this phenomenon of breeding season reduction might be already occurring (see figure S2). On the other hand, climate change will have a positive effect on breeding season length and stability towards the northern and eastern borders of rabbit range.

In addition, we evidenced that these reproductive modifications will likely cause strong and contrasting consequences at the level of population dynamics in the future. Climate change, through its impact on breeding season, will lead to density declines, increased extinction probabilities, and, most importantly, lower resilience to perturbation in populations of south-western Europe, especially in the essential native populations of the Iberian Peninsula. On the contrary, populations in the northern and eastern countries will tend to increase and improve their recovery capacity. The here demonstrated high importance of breeding period on rabbit population dynamics confirms the results of other authors showing that in small mammals with early sexual maturation and large litter size, reproductive parameters have a relatively high impact on population growth [42], [43]. These results are especially interesting in the case of European rabbits since, despite being considered a fast life-history mammal [11], most authors have usually focused only on survival rates and mortality causes to explain their conservation or pest status.

It is still uncertain how these climate-induced changes in reproduction, will ultimately affect dynamics and distribution of real rabbit populations, since we lack a clear picture of how climate will simultaneously alter survival patterns, by relaxing harsh cold winters, increasing droughts and floods, changing carrying capacities and modifying the impact and phenology of infectious diseases and all these other factors have been previously showed to be determinant for rabbit population dynamics (see reference [14] and [15] for extensive reviews). However, the aim of this study was not to predict absolute number or changes in rabbit populations across Europe, but rather our intention was to understand the complexity of impacts that climate change can cause just by altering some of the parameters determining species population dynamics. Nonetheless, the strong relationship found between current rabbit occupancy in Europe and reproductive periods predicted for 1961–1990, with breeding season length alone classifying correctly the presence/absence of rabbits in over 80% of the map cells (see Text S1), suggests that rabbit distribution will likely follow similar trends to those found in this study. That is, all the other parameters remaining within similar ranges of values, future patterns in breeding season will lead to shifts in rabbit dynamics and distribution towards more northern latitudes (Scandinavia), eastern regions (the Balkans), and higher altitudes (the Alps).

These results do not necessarily mean that native rabbit populations (SW Europe) will disappear completely since the greater genetic diversity in these populations [44] could allow them to adapt to environmental changes. Besides, the scale mismatching prevents us also from inferring the fate of specific populations directly from our regional predictions since there may be local factors, such as water bodies, crops, altitudinal gradients, specific hunting management or disease dynamics, determining population-specific breeding seasons and survival rates. Thus, although we acknowledge that in order to make more reliable predictions about particular populations future studies should consider site-specific scenarios with more detail information about factors such as mortality causes and epidemiological dynamics, at the same time we can affirm that the decrease in length and increase in variability of the rabbit breeding season in these southern areas will certainly have a detrimental impact in those populations.

In conclusion, wild rabbit productivity and populations will most likely respond to climate change differently across space emphasizing even farther the conservation-pest dualism of this species. The desertification of SW Europe will negatively affect rabbit populations within their native range, where they are a keystone species already in decline [45], [46]. This could lead to important cascading effects on Mediterranean communities and predators relaying on them. Contrarily, in the north and east of Europe, populations will be enhanced and new adjacent areas might be colonized by rabbits. This will have negative consequences for native biota in those areas, including potential invasional meltdown as already demonstrated in Chile, where exotic rabbits facilitate plant invasions [47]. In the rest of their European distribution, breeding season changes, may also lead to decreases in wild rabbit numbers, although the consequences of these declines will be less dramatic than in their native range since these introduced populations are currently thriving. Rabbit reductions in those areas may be beneficial due to alleviating the pressure of exotic rabbits on local biodiversity and agriculture; however, they may be also negative if they disrupt the new assembled communities [48].

As mentioned before climate change may have simultaneous effects on other demographic variables (e.g. survival), which are not considered here and that might be important, especially at the local level. Thus, more research will be needed in order to also explore the consequences of potential climate-driven changes in those parameters before making more specific predictions. However, this should not invalidate the general patterns found in this study showing the contrasting effects of climate change on large-scale population dynamics through reproduction. Moreover, this study is highly valuable since it abandons the non-mechanistic “future snapshots” produced by climatic envelopes and other type of projections directly based on current distribution patterns, and identifies the type of mechanisms used by climate change to modify species dynamics and ranges. Research like this that focus on the processes and not the patterns will help not only to better understand and predict the complex ways in which a species may respond to climatic alterations, but also to identify which are the main population parameters upon which we can concentrate our efforts in order to mitigate or compensate the effects of climate.

## Supporting Information

Figure S1 Control and future breeding season trends in Europe.
(DOC)

Figure S2 Past breeding season trends in SW Europe (Lisbon).
(DOC)

Table S1 Comparison of observed and predicted breeding season lengths.
(DOC)

Text S1 Association between distribution and European wild rabbit breeding season.
(DOC)

Text S2 Supporting references
(DOC)
