# Supplementary material for: Contrasting Effects of Climate Change on Rabbit Populations through Reproduction
Source: PLoS One. 2012 Nov 13;7(11):e48988. doi: 10.1371/journal.pone.0048988 (PMC3496743; doi:10.1371/journal.pone.0048988)
Supplement: Figure S1 — Control and future breeding season trends in Europe. (DOC) [file pone.0048988.s001.doc]

**
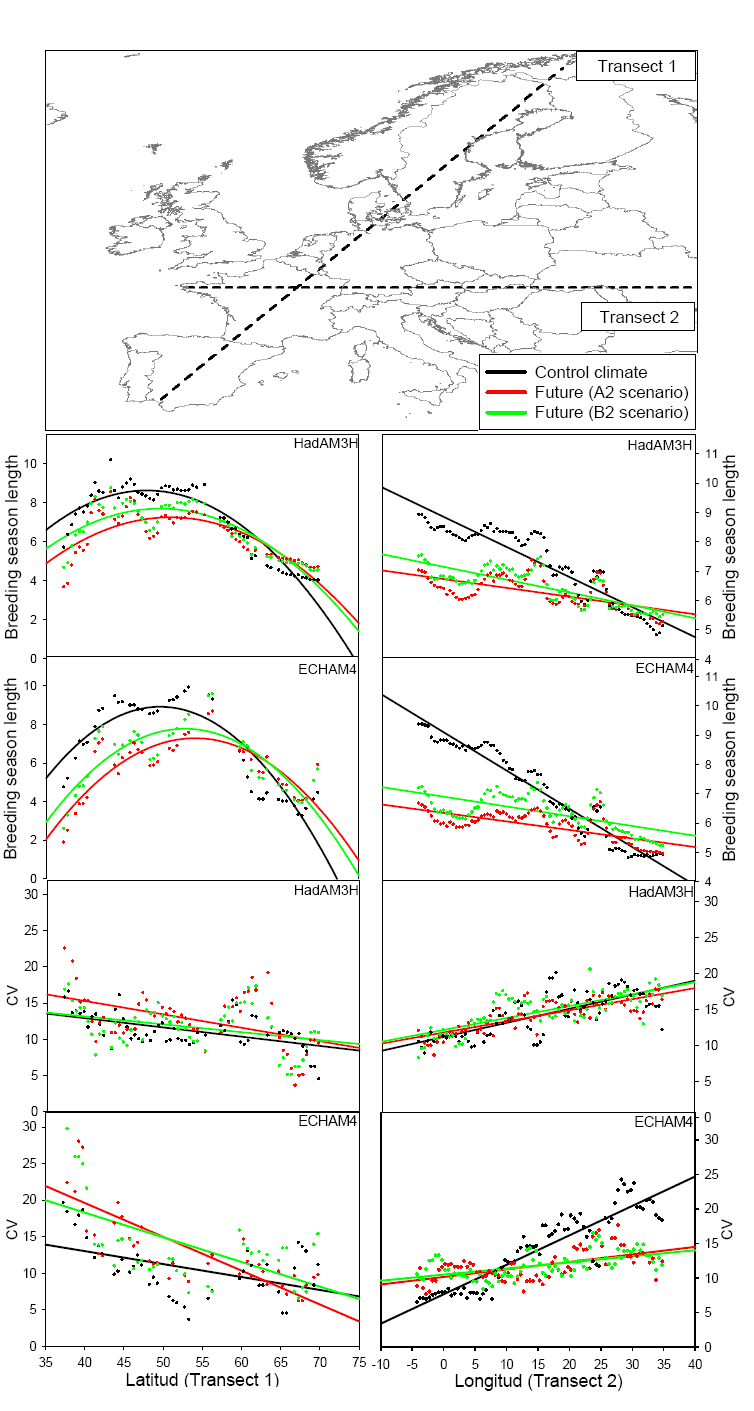
**

**Figure S1. *Control and future breeding season trends in Europe.*** Latitudinal and longitudinal trends in length and CV of rabbit reproductive period following two different transects across Europe for 1961-1990 (Control climate) and 2071-2100 (Future).Future breeding conditions were projected using two general circulation models (HadAM3H and ECHAM4/OPYC ) and a high (A2 scenario) and a moderate (B2 scenario) greenhouse gas emission scenarios. Points in the graphs represent breeding season lengths and CV predicted by the model of ref. 10 for each transect pixel, while lines added over them correspond to the quadratic or linear regression curves adjusted to them.
