# Supplementary material for: Contrasting Effects of Climate Change on Rabbit Populations through Reproduction
Source: PLoS One. 2012 Nov 13;7(11):e48988. doi: 10.1371/journal.pone.0048988 (PMC3496743; doi:10.1371/journal.pone.0048988)
Supplement: Figure S2 — Past breeding season trends in SW Europe (Lisbon). (DOC) [file pone.0048988.s002.doc]

**Figure S2. *Past breeding season trends in SW Europe (Lisbon).*** The top figure represents predicted breeding season trends by applying the mechanistic model in ref. 10 to Lisbon climatic data from 1870-2007. Small blue dots correspond to annual breeding season lengths while larger black and red dots are mobile estimations (in 30 year intervals) of duration and inter-annual variability of the reproductive period. The straight lines associated by colour to each group of points represent the linear trends of those parameters over time. The bottom image shows the phenological changes in rabbit breeding season in Lisbon, with reductions in probability of reproduction being more pronounced around summer months (July, October, and November).
