# Supplementary material for: Contrasting Effects of Climate Change on Rabbit Populations through Reproduction
Source: PLoS One. 2012 Nov 13;7(11):e48988. doi: 10.1371/journal.pone.0048988 (PMC3496743; doi:10.1371/journal.pone.0048988)
Supplement: Table S1 — Comparison of observed and predicted breeding season lengths. (DOC) [file pone.0048988.s003.doc]

**Table S1. *Comparison of observed and predicted breeding season lengths.*** Duration (months/year) of the reproductive period found in the literature (Observed) and predicted (mean ± standard deviation) using two general circulation models (HadAM3H and ECHAM4/OPYC ) for the period 1961-1990.

| **Location** | **Latitud** | **Longitud** | **Year** | **Breeding season** | | | **Source** |
| --- | --- | --- | --- | --- | --- | --- | --- |
|  |  |  |  | **Observed** | **HadAM3H** | **ECHAM4/OPYC** |  |
| Beja | 38.02 | -7.87 | 1976 | 8 | 6.54 ± 0.95 | 5.29 ± 0.89 | Ref. 1 |
| Alicante | 38.34 | -0.48 | 1994-97 | 7* | 7.14 ± 1.17 | 5.79 ± 1.13 | Ref. 2 |
| Sierra Morena | 37.90 | -6.38 | 1976-77 | 7 | 6.83 ± 1.02 | 5.57 ± 0.96 | Ref. 3 |
| Auffargis | 48.65 | 1.83 | 1977-79 | 7 | 8.18 ± 0.97 | 8.67 ± 0.69 | Ref. 4 |
| Caernarvonshire | 53.30 | -4.50 | 1941-42 | 6* | 9.79 ± 0.48 | 9.97 ± 0.18 | Ref. 5 |
| Doñana | 37.10 | -6.44 | 1974-75 | 5 | 5.7 ± 0.88 | 4.85 ± 0.9 | Ref. 6 |
| Camargue | 43.70 | 4.10 | 1975-77 | 4 | 8.84 ± 0.98 | 8.53 ± 0.86 | Rogers (1979) in Ref. 7 |
| Scania | 55.85 | 13.41 | 1972-76 | 6 | 8.26 ± 0.96 | 8.31 ± 1.06 | Ref. 8 |
| Kent | 51.19 | 0.74 | 1958-60 | 8* | 8.93 ± 0.94 | 9.45 ± 0.67 | Mead and Briggs (1977) in Ref. 9 |
| Navarra | 42.62 | -1.61 |  | 8* | 9.44 ± 0.92 | 8.86 ± 1.04 | Ceballos unpub. in Ref. 7 |
| ** years of study are outside the control climate period (1961-1990)* | | | | | |  |  |
