# Supplementary material for: Contrasting Effects of Climate Change on Rabbit Populations through Reproduction
Source: PLoS One. 2012 Nov 13;7(11):e48988. doi: 10.1371/journal.pone.0048988 (PMC3496743; doi:10.1371/journal.pone.0048988)
Supplement: Text S1 — Association between distribution and European wild rabbit breeding season. (DOC) [file pone.0048988.s004.doc]

**Text S1: Association between distribution and European wild rabbit breeding season**

We performed generalized linear models to relate the distribution of the wild rabbit in Europe to predicted breeding seasons for 1961-1990. As the dependent variable we used the presence or absence of rabbits (binomially distributed). According to the data on rabbit distribution provided by ref. 11, we assigned the value one (occurrence of rabbits) or zero (absence of rabbits) to every point in a grid of 50km resolution over Europe.

We then tested if the predicted mean length and inter-annual variability (CV) in the reproductive period and the interaction between both variables could explain to some extent that distribution through a logistic regression. Breeding seasons used were the average between the results of applying the model in ref. 9 to climate data from the two different general circulation models (i.e. HadAM3H and ECHAM4/OPYC).

Although the CV or its interaction with duration were not found to be significant, breeding season length showed a strong positive correlation with rabbit distribution (F = 922.43; p < 0.0001, Goodness of fit = 1.0). In fact, this variable alone explains more than 40 % of the deviance in rabbit distribution.
