# Supplementary material for: Contrasting Effects of Climate Change on Rabbit Populations through Reproduction
Source: PLoS One. 2012 Nov 13;7(11):e48988. doi: 10.1371/journal.pone.0048988 (PMC3496743; doi:10.1371/journal.pone.0048988)
Supplement: Text S2 — Supporting references (DOC) [file pone.0048988.s005.doc]

**Text S2: Supporting references:**

1. Ribeiro OL (1983) Quelques donnés sur la biologie du lapin de garenne (Oryctolagus cuniculus) au Portugal (Contenda – Sudest de Portugal). In: *XVth Congreso International de Fauna Cinegética y Silvestre* (ed. Caza EBdDaFEd) Trujillo 1981. pp. 607-613.
2. Arques J (2000) *Ecology and hunting management of a population of wild rabbits in ‏he south of the province of Alicante* (Translated from Spanish). PhD thesis (University of Alicante, Alicante, Spain).
3. Soriguer RC (1981) *Biology and dynamics of a population of rabbits (Oryctolagus cuniculus, L.) in Occidental Andalusia* (Translated from Spanish). Doñana Acta Vertebrata 8: 1-379.
4. Arthur CP (1980) Demography of wild rabbit (*Oryctolagus cuniculus (L.)* 1758) in an area of Paris (Translated from French). Bull. Mens. Office National de la Chasse. Numero special scientifique et technique: 127-162.
5. Brambell FWR (1944) The reproduction of the wild rabbit, *Oryctolagus cuniculus*. Proc. Zool. Soc-London 114: 1-45.
6. Delibes M, Calderon J (1979) Data on rabbit reproduction, Oryctolagus cuniculus (L.), in Doñana, SW of Spain, during a dry year (Translated from Spanish). Doñana Acta Vertebrata 6: 91-99.
7. Rogers PM, Arthur CP, Soriguer RC (1994) The rabbit in continental Europe. In: The European rabbit: the history and biology of a successful colonizer*.* (eds. King HVTaCM). Oxford University Press. Oxford, UK. pp. 22-62.
8. Andersson M, Meurling P, Dahlback M, Jansson G, Borg B (1981) Reproductive biology of the wild rabbit in southern Sweden, an area close to the northern limit of its distribution. In: Proceedings of the World Lagomorph Conference (ed. MacInnes. KMaCD) Guelph, Ontario, 1979. pp. 175-181.
9. Thompson HV (1994) The rabbit in Britain. In: The European rabbit: the history and biology of a successful colonizer. (eds. King HVTaCM). Oxford University Press Oxford, UK. pp. 64-107.
10. Tablado Z, Revilla E, Palomares F (2009) Breeding like rabbits: global patterns of variability and determinants of European wild rabbit reproduction. Ecography 32: 310-320.
11. Mitchell-Jones AJ, Amori G, Bogdanowicz W, Krystufek B, Reijnders PJH, et al. (1999) Atlas of European Mammals. Academic Press, London.
